# Chronic Hepatitis E Infection Resulting in Graft Failure in a Liver Transplant Tourist

**DOI:** 10.1155/2011/654792

**Published:** 2011-07-02

**Authors:** Hui-Hui Tan, Hoe-Nam Leong, Boon-Huan Tan, Lynette Lin-Ean Oon, Kiat-Hon Lim, Jason Pik-Eu Chang, Chee-Kiat Tan

**Affiliations:** ^1^Liver Transplant Service, Department of Gastroenterology & Hepatology, Singapore General Hospital, Singapore 169608; ^2^Department of Infectious Diseases, Singapore General Hospital, Singapore 169608; ^3^Defence Science Organisation, Singapore 118230; ^4^Department of Pathology, Singapore General Hospital, Singapore 169608

## Abstract

Hepatitis E, usually an acute hepatitis in the immunocompetent, has a chronic form described in immunocompromised hosts. We report the clinical course and outcome of an adult liver transplant recipient whose posttransplant period was complicated by chronic hepatitis E, Epstein-Barr virus infection, and cellular rejection of the graft.

## 1. Introduction

Hepatitis E virus (HEV) infection is known to be transmissible via various routes. A chronic form of persistent HEV has been described in immunocompromised hosts, especially in transplant recipients who are on immunosuppression. We describe an adult liver transplant (LT) recipient whose post-LT course was complicated by chronic HEV, Epstein-Barr viremia (EBV), and acute cellular graft rejection resulting in management dilemmas and ultimately graft failure and mortality.

## 2. Case Report

Mr. X was a 48-year-old Chinese male with chronic hepatitis B virus infection and multifocal hepatocellular carcinoma that was beyond standard criteria, precluding LT. In early November 2009, he approached a center in another country as a transplant tourist and received a deceased liver graft. Postoperatively, he was discharged with tacrolimus 1mg bid, mycophenolate mofetil 750 mg bid, and prednisolone 20 mg od. 

**Figure 1:**
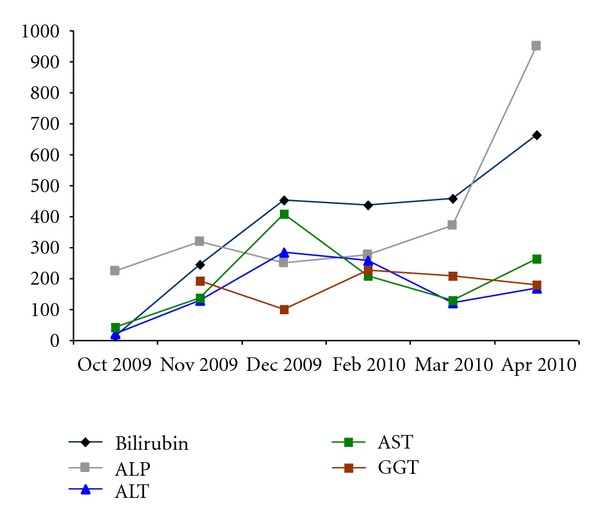
Serial liver tests for patient Mr. X. Bilirubin (Normal: 3–24 umol/L), ALP: Alkaline phosphatase (Normal: 32–103 U/L), ALT: alanine transaminase (Normal: 7–36 U/L), AST: aspartate transaminase (Normal: 15–33 U/L), GGT: *γ*-glutamyl transferase (Normal: 11–63 U/L).

He returned to us 3 weeks later to continue post-LT care at our center. He was noticed to be deeply jaundiced with a mixed cholestatic-hepatitic picture on his liver tests (see Table 1 and Figure 1). 

Serology and blood polymerase chain reaction (PCR) were positive for Epstein-Barr virus (EBV) and hepatitis E virus (HEV) genotype 3. Liver tissue was also positive for EBV by PCR. Serology and blood PCR were negative for cytomegalovirus, herpes simplex virus, hepatitis B virus, hepatitis C virus, parvovirus, and human herpes virus 6. Liver tissue was negative for viral inclusions. He was treated with acyclovir for EBV infection with demonstrable reduction in serum EBV viral load by PCR. 

**Figure 2:**
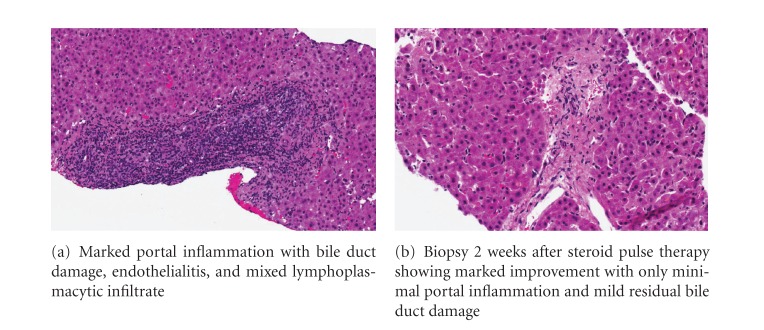
Histomicrograph of liver biopsy in December 2009.

**Figure 3:**
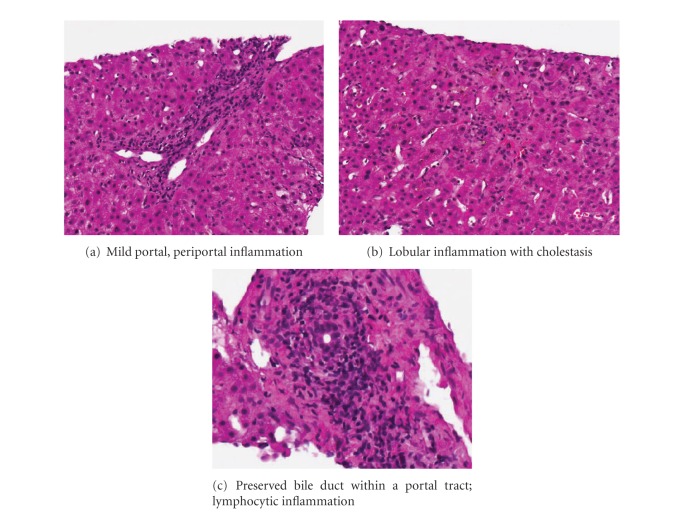
Histomicrograph of liver biopsy in February 2010.

Doppler imaging of the graft confirmed patent graft vessels. Magnetic resonance imaging was suggestive of an anastomotic biliary stricture, and this led to successful endoscopic deployment of a biliary stent across the stricture. However, despite regular stent changes per 3 months with good bile outflow, his liver tests did not improve and he remained deeply jaundiced. A liver biopsy was performed in December 2009 that demonstrated moderate acute cellular rejection (rejection activity index score 5) (Figure 2(a)). This was treated with pulse methylprednisolone. Repeat biopsy performed 2 weeks later confirmed good histological response of the acute cellular rejection to steroid pulses (Figure 2(b)). However, liver function tests did not improve, and a third liver biopsy in February 2010 demonstrated only lymphocytic portal and lobular hepatitis with marked cholestasis, without acute or chronic graft rejection (Figure 3). Metavir fibrosis score was 1/4. No atypical mononuclear infiltrate was seen in the biopsy specimen. There was no evidence of lymphoproliferative disease in any of the liver histologies nor on serial radiologic imaging. 

His graft function continued to deteriorate with HEV RNA detectable up to 6 months after LT. He was admitted in April 2010 with jaundice, ascites, peripheral oedema, and constitutional symptoms. Graft failure with disseminated bacterial and fungal infection led to his demise soon after (6 months post-LT).

## 3. Discussion

HEV is an RNA virus, transmissible by the fecal-oral route, usually through contaminated food and water. However, it is now recognized that in the viremic phase, HEV transmission may be blood borne as well [1–5]. It is endemic in several developing countries [6], but not in Singapore. Phylogenetic analysis classifies HEV into 4 genotypes [7, 8]. Genotype 1 is the most prevalent and widespread. Genotype 2 has been reported in Mexico and West Africa. Genotype 3 has been isolated from nonendemic regions such as the United States, Europe, and Japan. Genotype 4 is less commonly isolated and has been found in specimens from China, Taiwan, Japan and Vietnam. HEV genotypes 1 and 2 primarily infect humans, whilst genotypes 3 and 4 have been isolated in swine and other animals in areas where human isolates have been found [9]. Reverse zoonotic transmission has also been reported [10].

Although previously assumed to manifest only as an acute hepatitis with spontaneous resolution, recent data and literature now suggest that HEV infection does have a chronic form, more commonly seen in the immunocompromised host. Most reports of chronic HEV have been in transplant recipients on immunosuppression. CD4+ IFN*γ*-secreting cells and natural killer T cells may be involved in the pathogenesis of HEV [11]. The greater severity of hepatitis E infection in pregnant women suggests a relation to the Th2 response [12]. Solid-organ transplant recipients with chronic hepatitis E have been found to have significantly lower total lymphocyte and CD2, CD3, and CD4 subset counts as opposed to recipients who had cleared the HEV [13]. Interestingly, HEV reactivation has also been reported in a patient after stem cell transplantation [14]. Hence, it is probably under these circumstances that nonpersistent viruses may result in chronic colonization and recurrent infection [15]. Chronic HEV infection may induce a rapid and severe liver disease, ranging from protracted HEV infection to chronic active hepatitis to progressive cirrhosis [16–18]. 

The diagnosis of HEV infection is usually made on the basis of positive serology in the immunocompetent host. However, in the context of immunosuppression, antibody testing has been reported to be repeatedly negative despite detectable HEV RNA in the blood [19]. Transplant clinicians should be mindful of this, and molecular testing should be performed if the index of suspicion is high, regardless of serological results. 

Our case report is interesting for the following reasons. 

Firstly, our patient returned from the overseas centre 3 weeks after transplant with a persistent, progressive liver dysfunction which did not improve despite successful biliary stenting and treatment for graft rejection. HEV serology was positive within 5 weeks post-LT. To our knowledge, our patient is one of the earliest to be diagnosed with HEV infection so soon after LT. As HEV genotype 3 is of the swine variety, it is possible that the patient had acquired it from ingestion of contaminated pork. However, it could also have been the result of implantation of an organ from a cadaveric donor who previously had HEV infection, resulting in reactivation with immunosuppression. HEV serology was not tested in our patient prior to LT as there had been no clinical indication to do so. How our patient would have acquired HEV and if transplant tourism increased the risk for this, we are unable to prove in this recipient. HEV is not endemic in our country. However, the implications of transplant tourism include having broad differentials for the cause of graft hepatitis. In light of the scarcity of available organs and the rising costs of healthcare, transplant clinicians should be mindful to consider unusual/uncommon infections from their patients returning from other centers. 

Secondly, our patient also had concurrent EBV infection as demonstrated by serology and detectable DNA in the blood and liver tissue. EBV infections are commonly observed in patients after liver transplantation, and serologic evidence of active infection is demonstrable in close to 25% of cases [20]. This is usually asymptomatic disease. However, 1-2% of liver transplant recipients may have persistent or recurrent disease resulting in the development of posttransplant lymphoproliferative disorders (PTLDs) and lymphomas [21]. Although our patient did not demonstrate the classical features of infectious mononucleosis-like EBV infection, the decision was made to commence acyclovir treatment in view of persistent hepatitis and demonstrable EBV DNA in the blood and liver tissue. It is possible that this patient's EBV infection was a red herring, as graft function continued to deteriorate despite EBV treatment which had some documented response (one log drop in viral load). Furthermore, death or graft failure reported from EBV is commonly in the context of PTLD, rather than from viral infection per se. On the other hand, despite recent reports of HEV infection in solid-organ transplant recipients, most were not described to have concurrent EBV infection as well. Except for the report of a kidney-pancreas transplant recipient by Kamar et al., who developed acute hepatitis from HEV 6 months after transplant and who also had detectable EBV DNA in the blood, we are not aware of other reports of patients with concurrent EBV-HEV infection [18]. EBV was not demonstrable by immunohistochemistry in the liver tissue of Kamar et al.'s patient, and the EBV DNA level in the blood was relatively unchanged both before and after the acute hepatitis episode (4.3 log_10_ copies/mL). In their case report, a liver biopsy performed 16 months from the diagnosis of HEV infection demonstrated moderate hepatitis with a Metavir fibrosis score of F2.22 months from the diagnosis of HEV infection; their patient was cirrhotic and soon after succumbed to a bacterial infection. It is possible that EBV hepatitis or infection contributed to or aggravated the rapid progression of HEV in our patient. After all, both retrospective and prospective data from India and China suggest that superimposed HEV infection on patients with chronic liver hepatitis or liver disease has a poorer prognosis [22–24]. Whether EBV infection or reactivation contributes to hepatitis with a poor outcome in recipients with HEV infection should be explored in future studies, as positive EBV serology is not uncommon in solid organ transplant recipients.

Thirdly, as inferred from the pathogenesis of chronic HEV above, reducing immunosuppression that targets T cells may be a possible therapeutic option [18]. However, cellular rejection in our patient's graft necessitated steroid pulses and also made it difficult to tail immunosuppressants early. Recent reports suggest that pegylated interferon-*α*-2a/2b may have a role in the treatment of liver transplant recipients with chronic HEV in whom the lowering of immunosuppressant doses alone is insufficient [25, 26]. However, optimal treatment duration and the risk of associated graft rejection with interferon therapy are yet to be determined in this patient population. Kamar et al.'s case series of 3 liver transplant recipients treated with pegylated interferon-*α*-2a for chronic HEV infection reported acute humoral rejection in the liver biopsy of one of their patients after 12 weeks of therapy. Fortunately for that patient, HEV RNA was undetectable by then and remained so, despite steroid pulses required to treat graft rejection [26]. 

Transplant recipients should be cautioned against consuming meat from partially cooked animal sources which may harbor the HEV. Although a recent large, phase 3 study has demonstrated efficacy of an HEV recombinant vaccine [27], more studies would be required before formal recommendations can be made on whether HEV vaccination should be offered to all potential transplant candidates prior to transplantation, especially in endemic areas, or whether HEV serology testing should be performed pre-LT, assuming its positive implications for reactivation in a potential transplant candidate.

Transplant tourism may be an enticing option for the deteriorating LT candidate waiting at length for a suitable graft. Even some global medical insurance programs are promoting this option [28]. However, it is fraught with both clinical and public health implications. Apart from the standard of hygiene and quality of postoperative care in overseas centers, recipients have been reported to have contracted infections endemic to overseas centers (e.g., tuberculosis, aspergillus, and hepatitis B) or even donor malignancies [29–31]. Medical records from overseas centers are often incomplete, scant, in a foreign language, or unobtainable [29, 32, 33]. Fatal postoperative bleeding has also been reported, as have both under or over-immunosuppresion, and infection with the HIV virus [29, 30, 32, 34, 35]. Renal transplant tourists have been reported to have a more complex posttransplantation course with a higher incidence of graft rejection and severe infectious complications [36].

## 4. Conclusion

The transplant tourist presents a unique set of potential complications and problems not usually encountered in one's own center. The physician is required to consider a wide list of differentials and to think “out-of-the-box” when managing such recipients, especially in the first year after transplant.

## Figures and Tables

**Table 1a:** (a)

Date	Oct-09	24 Nov-09	28 Nov-09	4 Dec-09	12 Dec-09
Event	pre-OLT	OLT 3 Nov-09	ERCP 26 Nov-09	Liver biopsy	Liver biopsy 11 Dec-10 ERCP 11 Dec-10
Bilirubin	14	246	298	453	458
(Normal: 3–24 umol/L)					
Alkaline phosphatase	224	319	244	251	138
(Normal 32–103 U/L)					
Alanine transaminase	22	129	151	285	169
(Normal: 7–36 U/L)					
Aspartate transaminase	43	138	172	407	164
(Normal: 15–33 U/L)					
*γ*-Glutamyl transferase	—	192	126	100	77
(Normal: 11–63 U/L)					

**Table 1b:** (b)

Date	17 Dec-09	Feb-10	Mar-10	Apr-10
Event	ERCP 14 Dec-10	Liver biopsy	ERCP stent change	2 weeks prior to demise
Bilirubin	471	438	457	662
(Normal: 3–24 umol/L)				
Alkaline phosphatase	128	277	372	951
(Normal 32–103 U/L)				
Alanine transaminase	163	257	121	168
(Normal: 7–36 U/L)				
Aspartate transaminase	137	207	128	264
(Normal: 15–33 U/L)				
*γ*-Glutamyl transferase	67	227	207	180
(Normal: 11–63 U/L)				

## Abbreviations

HEV: Hepatitis E virus
EBV: Epstein-Barr virus
LT: Liver transplantation
PCR: Polymerase chain reaction
PTLD: Post-transplant lymphoproliferative disorders
